# Melatonin Mitigates Cisplatin-Induced Submandibular Gland Damage by Inhibiting Oxidative Stress, Inflammation, Apoptosis, and Fibrosis

**DOI:** 10.7759/cureus.68515

**Published:** 2024-09-03

**Authors:** Alaa M Badawy, Mohie Ibrahim, Medhat Taha, Azza I Helal, Ahmed Abdel-Monem Elmetwally, Ibrahim El-Shenbaby, Sara Abubakr, Emadeldeen Hussin, Noha Hammad Sakr, Tourki A. S Baokbah, Amira E Farage

**Affiliations:** 1 Department of Anatomy and Embryology, Faculty of Medicine, Mansoura University, Mansoura, EGY; 2 Department of Basic Medical and Dental Sciences, Faculty of Dentistry, Zarqaa University, Zarqa, JOR; 3 Department of Anatomy, Umm Al-Qura University, Al-Qunfudhah, SAU; 4 Department of Histology and Cell Biology, Faculty of Medicine, Kafrelsheikh University, Kafr El Sheikh, EGY; 5 Department of Clinical Pharmacology, Faculty of Medicine, Mansoura University, Mansoura, EGY; 6 Department of Anatomy and Embryology, Faculty of Medicine, Kafrelsheikh University, Kafr El Sheikh, EGY; 7 Department of Medical Emergency Services, Umm Al-Qura University, Al-Qunfudhah, SAU

**Keywords:** apoptosis, fibrosis, submandibular gland, inflammation, oxidative stress, cisplatin, melatonin

## Abstract

Background: The study aims to examine the possible effect of melatonin against cisplatin-induced submandibular degeneration in experimental rats exploring its ameliorative mechanisms.

Methods: Rats were classified into four experimental groups; control group; melatonin group; cisplatin group; and cisplatin+melatonin group. Submandibular tissues were collected. Biochemical, histopathological, and immunohistopathological examination and quantitative reverse transcription polymerase chain reaction (qRT-PCR) analysis were performed.

Results: The results indicate that intraperitoneal administration of melatonin (30 mg/kg body weight) alongside cisplatin significantly elevated submandibular glands (SMG) and reduced glutathione (GSH) and superoxide dismutase (SOD) levels (p < 0.001), while it reduced malondialdehyde (MDA) levels, NF-κB gene expression, the protein level of tumor necrosis factor-alpha (TNF-α), interleukin-6 (IL-6), interleukin-1 beta (IL-1β), immunoexpression of low-dose cyclooxygenase-2 (Cox-2), and CD68. Moreover, melatonin reduced immune and gene expression of alpha-smooth muscle actin (α-SMA), immunoexpression of caspase-3, and gene expression of Bax in comparison to the cisplatin group.

Conclusion: Melatonin attenuated cisplatin-induced submandibular destruction alleviating SMG oxidative stress, inflammation, and fibrosis in addition to halting cellular apoptosis, sheds light on its usage in clinical application.

## Introduction

Chemotherapy is commonly employed as the main treatment for certain cancers, as a palliative measure for advanced tumors, and as an adjunct therapy alongside local treatments such as surgery and radiotherapy. The primary objectives of chemotherapy are to eradicate metastases and enhance the management of local tumors [1,2]. However, chemotherapy can also damage healthy cells and tissues in addition to targeting cancer cells. The specific therapy, dosage, and duration of treatment influence the extent of its cytotoxic effects [3].

Cisplatin (cis-diamminedichloroplatinum(II)) is a very powerful drug used in treating multiple malignancies. Its mechanism of action is through forming DNA adducts and DNA damage, which in turn cause cell cycle arrest and apoptosis in cancer cells [4]. Despite its effectiveness, cisplatin is associated with numerous adverse effects, including neurotoxicity, ototoxicity, and renal toxicity. It also impacts the submandibular and parotid salivary glands, causing oral issues like mucositis (stomatitis), xerostomia (dry mouth), bleeding tendencies, dental caries, and loss of taste, which can increase the risk of oral infections. Reactive oxygen species (ROS) generation and lipid peroxidation, producing oxidative tissue destruction, are the cause of cytotoxicity [5,6].

The pineal gland in animals and humans is the primary source of melatonin (N-acetyl-5-methoxytryptamine) through synthesis and secretion [7]. This compound exhibits potent antioxidant and anti-inflammatory properties, which contribute to maintaining oral homeostasis [8,9]. The presence of melatonin in saliva can be attributed to the passive diffusion of unbound melatonin from blood plasma into the salivary glands. However, studies on animals and humans have also identified enzymes involved in melatonin synthesis within the ductal cells and the oral lingual mucosa, denoting that melatonin is synthetized in ducts excreting saliva and the oral cavity. Furthermore, the salivary glands can store and secrete melatonin. This is evidenced by the presence of granules and vesicles in different cells of salivary gland ducts [10]. Consequently, melatonin functions against inflammation and oxidative damage in oral tissues [8-10]. The present study was planned to detect the harmful effects of cisplatin on the submandibular salivary gland in vivo and to analyze the potential protective mechanisms of melatonin action.

## Materials and methods

Chemicals

Cisplatin was sourced from an Egyptian pharmacy in injection vials (50 mg/50 mL, Mylan, Italy). Melatonin powder, with a concentration of 50 mg melatonin per mL of ethanol, was obtained from Sigma Aldrich (St. Louis, MO, USA).

Experimental animals

Forty male adult Wistar rats, with a weight of 200-250 grams, were housed in groups of three on a 12-hour light/dark cycle, kept at a temperature of 24 ± 1°C, and fed a pellet diet. The study was conducted in accordance with the Canadian Council on Animal Care Guidelines and approved by the Committee of Research Ethics, Kafrelsheikh University (KFS-IACUC/204/2024).

Experimental design and sample collection

Four equal groups of 10 rats each were utilized in this study, as detailed in Table 1.

**Table 1:** Experimental groups

Experimental groups	Methods of drug administration
Control group	Rats received a mixture of normal saline and ethanol intraperitoneally one time per day for 10 days.
Melatonin group	Received melatonin with a dose of (30 mg/kg body weight; administered intraperitoneally) once daily for 10 days [11].
Cisplatin group	Received a single dose of cisplatin (5 mg/kg body weight; administered intraperitoneally) on the first day of the experiment [12].
Cisplatin+melatonin group	Received both drugs.

Rats of different groups anesthetized with 75 mg/kg sodium thiopental intraperitoneally on day 11. Rats were dissected and the submandibular gland (SMG) was excised. The right SMG was prepared for light microscopy, while the left SMG was flash-frozen and stored at -80°C for molecular and biochemical analysis.

Investigations

Preparation of Tissue Homogenates and Evaluation of Antioxidant Capacity Parameters

The dissected submandibular tissue was thoroughly washed with distilled water to remove any blood. Subsequently, the tissues were mixed in ice-cold sodium phosphate 50 mM (pH 7.4) containing 0.1 mM ethylenediaminetetraacetic acid (EDTA). Centrifugation of homogenates was done at 5000 rpm for 20 minutes at 4°C to separate the supernatant. Oxidative stress markers analyzed in the supernatant from SMG tissues included superoxide dismutase (SOD), catalase (CAT), reduced glutathione (GSH), and malondialdehyde (MDA). These markers were assessed using commercial kits (Biodiagnostics Co., Cairo, Egypt). The measurements were conducted with utmost care and precision using a spectrophotometer, following the factory instructions provided in the accompanying pamphlets.

Assessment of Inflammatory Markers

Tumor necrosis factor-alpha (TNF-α), interleukin-6 (IL-6), and interleukin-1 beta (IL-1β) levels were measured calorimetrically using commercial ELISA kits (Cat# KRC3011 for TNF, ERA31RBX5 for IL6, and ERIL1B for IL1β) (Bio-Rad Laboratories, Inc., Hercules, USA) following the manufacturer's instructions and guidelines.

Histopathological Examination

The specimens of the SMG were fixed in 10% formalin solution and subsequently rinsed under running tap water to get rid of any residual fixative. Following fixation, the specimens underwent dehydration by successive immersion in upgrading alcohol concentrations, followed by clearing in xylene. Once dehydrated, the samples were embedded in wax blocks of paraffin and positioned centrally. The blocks were then trimmed and sectioned into slices of 5-µm thickness. These sections were transferred through downgraded alcohol concentrations and distilled water before being stained with hematoxylin and eosin (H&E) for histological investigation [13].

Immunohistochemical Examination

Tissue sections, 4 µm-thick, were cut from paraffin blocks. Deparaffinization of the paraffin sections was achieved using xylene, followed by rehydration with decreasing concentrations of alcohol. Retrieval of antigen was done by heating the sections of submandibular tissue in a pressure cooker filled with sodium citrate buffer. The sections were then washed with phosphate-buffered saline (PBS) three times for five minutes after the blockage of the endogenous peroxidase activity was performed by 3% hydrogen peroxide in water. To block nonspecific immune reactions, the sections were incubated with a power block solution for 10 minutes at room temperature. Mouse monoclonal antibodies (anti-Cox2, anti-CD68, anti-α smooth muscle actin, anti-transforming growth factor beta (anti-TGFβ), and anti-caspase-3; Catalog# PA1-37505, MA5-16654, MA5-41117, MA5-16949, MA1-16843) were applied to the sections and incubated at room temperature (25ºC) for 60 minutes, followed by overnight incubation at 4ºC. Subsequently, the sections were counterstained with H&E. The amount of super-enhancer reagent used was determined based on the size of the tissue specimen to maximize the potency of the antigen-antibody reaction (38 µL). The sections were incubated with an appropriate volume of poly-horseradish peroxidase reagent at room temperature (25ºC) for 30 minutes and completely washed with PBS three times. After that, the sections were incubated with 3,3'-diaminobenzidine (DAB) solution for 30 minutes at room temperature (25ºC) to visualize the antigen-antibody reaction. After washing the sections with PBS at least three times, they were counterstained with Mayer’s hematoxylin and coverslipped using distyrene, polystyrene, and xylene [14]. Positive staining for the antigens was indicated by intranuclear brown coloration obtained from the DAB staining in the cells.

Quantitative Reverse Transcription Polymerase Chain Reaction (qRT-PCR) Analysis

According to Khamis et al. [15], approximately submandibular tissue (30mg) was utilized for RNA extraction using Trizol (Invitrogen; Thermo Fisher Scientific, Waltham, MA, USA). Extracted RNA quality was assessed by assessing the A260/A280 ratio using the NanoDrop™ ND-1000 Spectrophotometer (NanoDrop Technologies, Wilmington, DE, USA) with 1.5 µL of RNA. cDNA synthesis was done by the High-Capacity cDNA Reverse Transcription Kit (Applied Biosystems™, Waltham, MA, USA), following the manufacturer's protocol. Subsequently, primers were prepared according to the manufacturer's instructions for downstream applications. The prime sequences of the genes are discussed in Table 2.

**Table 2:** Forward and reverse primers sequence of targeted genes Bax: Bcl-2-associated X protein; TGF-β1: transforming growth factor beta 1; NF-κB: nuclear factor kappa B; α-SMA: alpha-smooth muscle actin

Primer	Sequence of the primer
Bax	Forward: 5′-GTT CGC CTT CAT TAT GGA CTGCC-3′ Reverse: 5′-ATA GCA CCC TGT TCC CGC AAAG-3′
TGF-β1	Forward: 5′-GGA CTC TCC ACC TGC AAG AC-3′ Reverse: 5′-GAC TGG CGA GCC TTA GTT TG-3′.
NF-κβ	Forward: 5′- GAA ATT CCT GAT CCA GAC AAA AAC -3′ Reverse: 5′- ATC ACT TCA ATG GCC TCT GTG TAG -3′
α-SMA	Forward: 5′-CGA TAG AAC ACG GCA TCA TCA C-3′ Reverse: 5′-GCA TAG CCC TCA TAG ATA GGC A-3′.
Glyceraldehyde-3-phosphatedehydrogenase	Forward: 5’-TGGATTTGGACGCATTGGTC-3’ Reverse: 5’-TTTGCACTGGTACGTGTTGAT-3’

Statistical analysis

Study variables were expressed as mean ± standard deviation. Data analysis was conducted using one-way analysis of variance (ANOVA), followed by Tukey’s test for comparisons between multiple groups. A p-value of less than 0.05 (p < 0.05) was considered statistically significant. Statistical analysis was performed with GraphPad Prism version 8.0.0 for Windows (GraphPad Software, San Diego, USA).

## Results

Effect of melatonin on SMG histology

**Figure 1:**
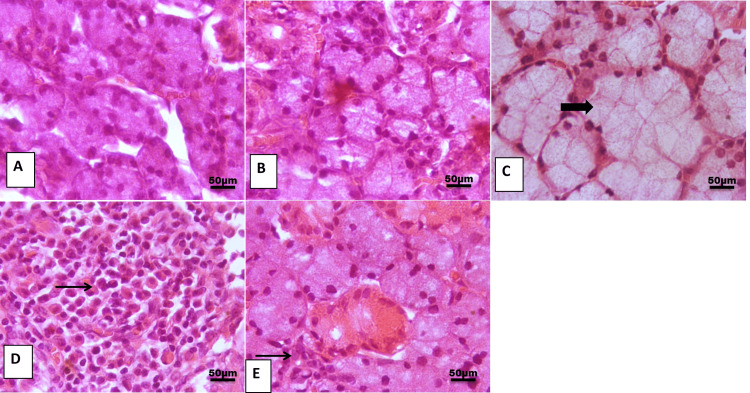
Representative photomicrograph of the submandibular gland from different treatment groups. (A, B) Control and melatonin group showing the normal histological architecture of glandular acini. (C) Cisplatin group showing marked dilation of mucous acini with pressure on interstitial connective tissue (thick arrow). (D) Cisplatin showing marked replacement of acinar architecture with cellular mass formed of abundant lymphoplasmacytic cells (thin arrow). (E) Cisplatin+melatonin showing focal peri-glandular aggregation of inflammatory cells (thin arrow). Image magnification: 400x

H&E examination of SMG of control and melatonin groups showing the normal morphology of SMG acini (Figures 1A, 1B). On the contrary, marked degeneration in the SMG of the cisplatin group was noted, including dilatation in mucosal acini with replacement of acinar architectures with inflammatory cell infiltration (Figures 1C, 1D). This histopathological change was restored by melatonin administration with minimal peri-glandular aggregation of inflammatory cells (Figure 1E).

Ameliorative effect of melatonin against cisplatin-induced SMG oxidative stress

**Figure 2:**
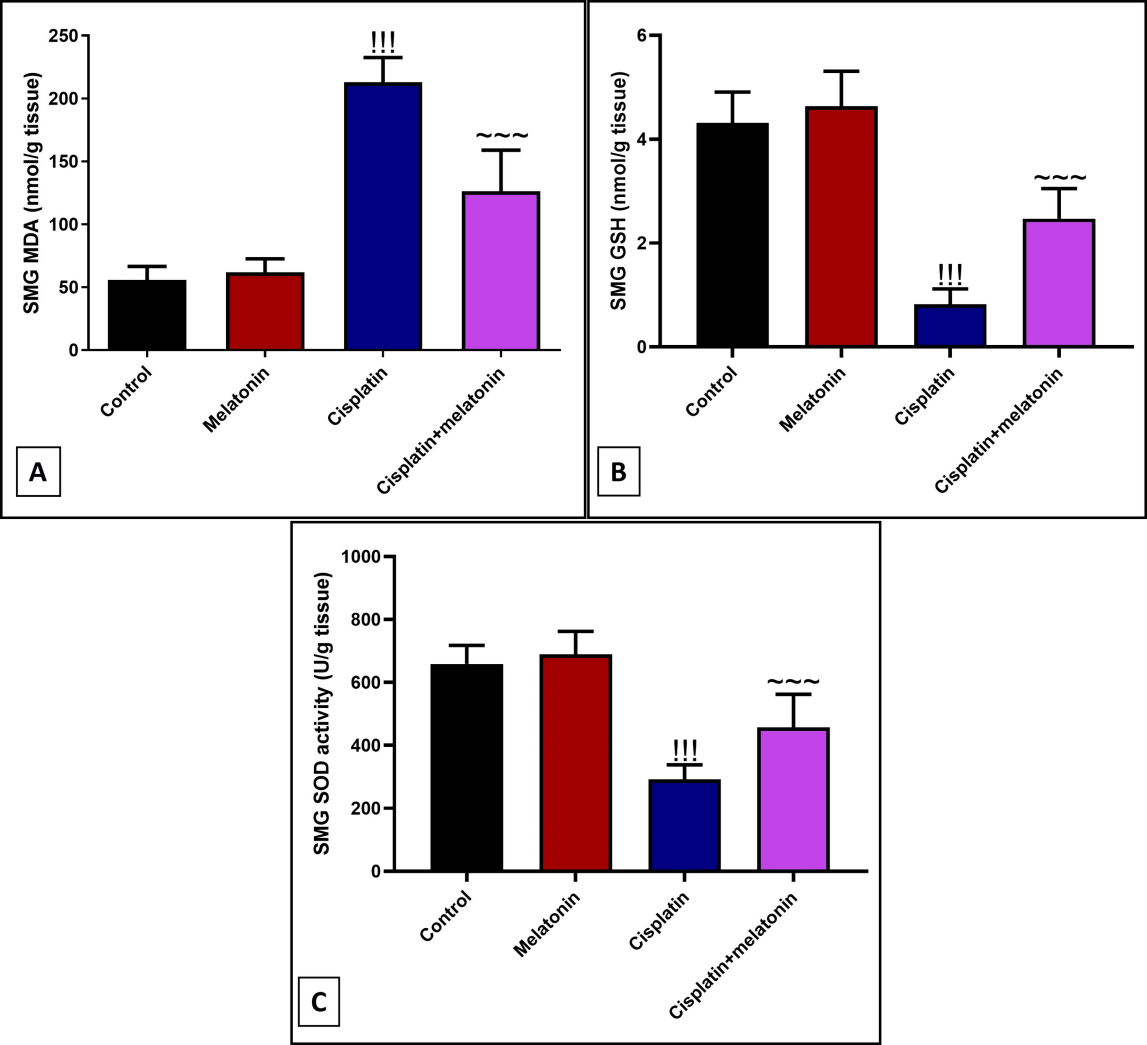
Markers of oxidative stress in submandibular glands of different experimental groups ((A) MDA, (B) GSH, and (C) SOD). Data were expressed in mean±SD. !!! indicates p < 0.001 significant versus control rats; ~~~ indicates p < 0.001 significant versus cisplatin group. MDA: malondialdehyde; GSH: glutathione; SOD: superoxide dismutase; SMG: submandibular gland

Intraperitoneal injection of cisplatin significantly (p < 0.001) increased submandibular lipid peroxidation marker MDA by 382% (Figure 2A), and decreased the levels of endogenous antioxidant GSH and SOD by 525.6% and 225.7% in comparison to control rats (Figures 2B, 2C). Melatonin intake significantly depressed MDA by 59.3%, and upregulates GSH and SOD by 301%, 156.6% compared to the cisplatin group.

Anti-inflammatory effect of melatonin against cisplatin-induced SMG inflammation

**Figure 3:**
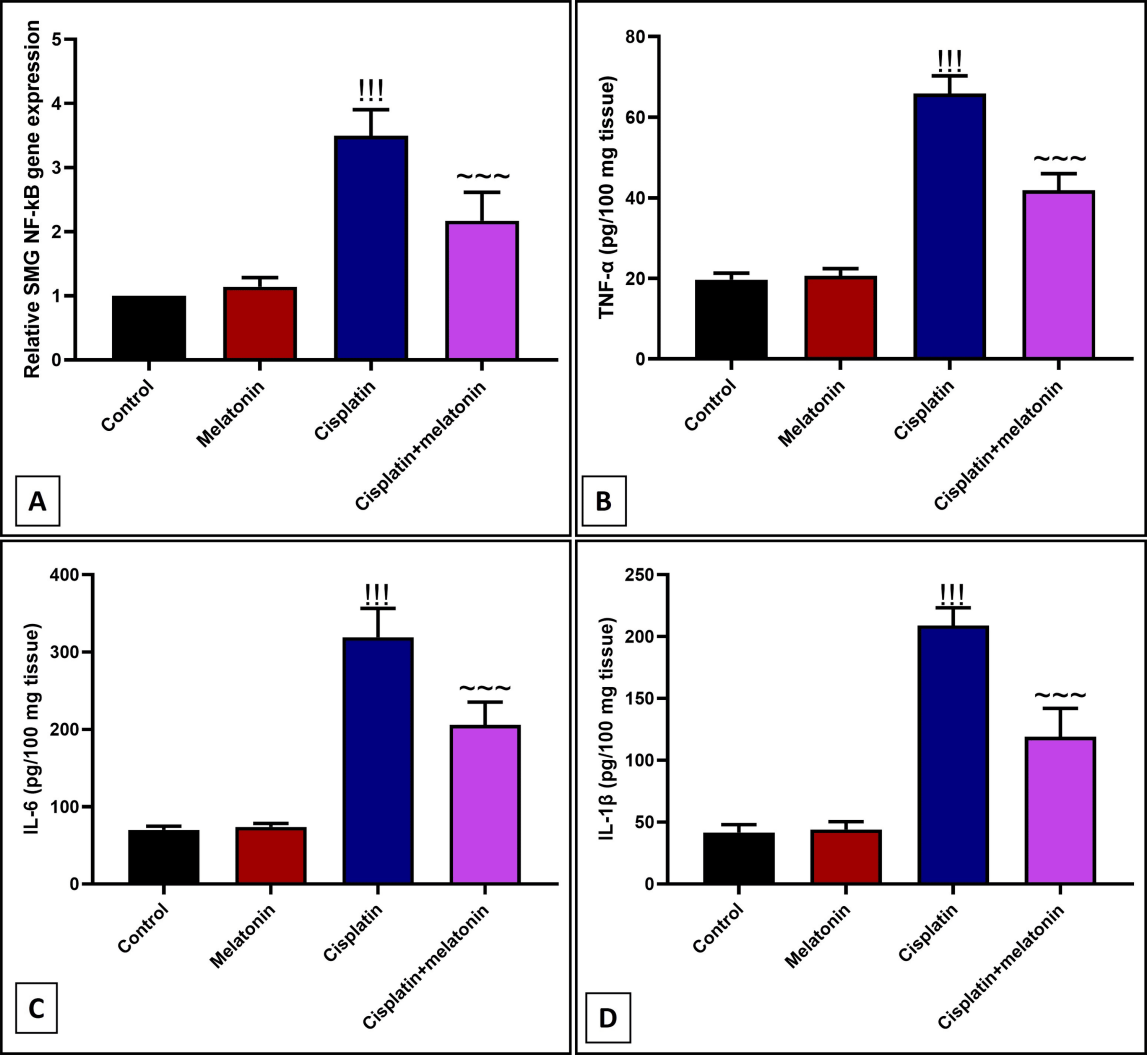
Effect of melatonin on cisplatin-induced inflammatory markers: (A) Gene expression of NF-κB. (B, C, D) Protein level of TNF-α, IL-6, IL-1β by Elisa assay. Data were expressed in mean±SD. !!! indicates p < 0.001 significant versus control rats; ~~~ indicates p < 0.001 significant versus cisplatin group. NF-κB: nuclear factor kappa B; TNF-α: tumor necrosis factor-alpha; IL-6: interleukin-6; IL-1β: interleukin-1 beta; SMG: submandibular gland

**Figure 4:**
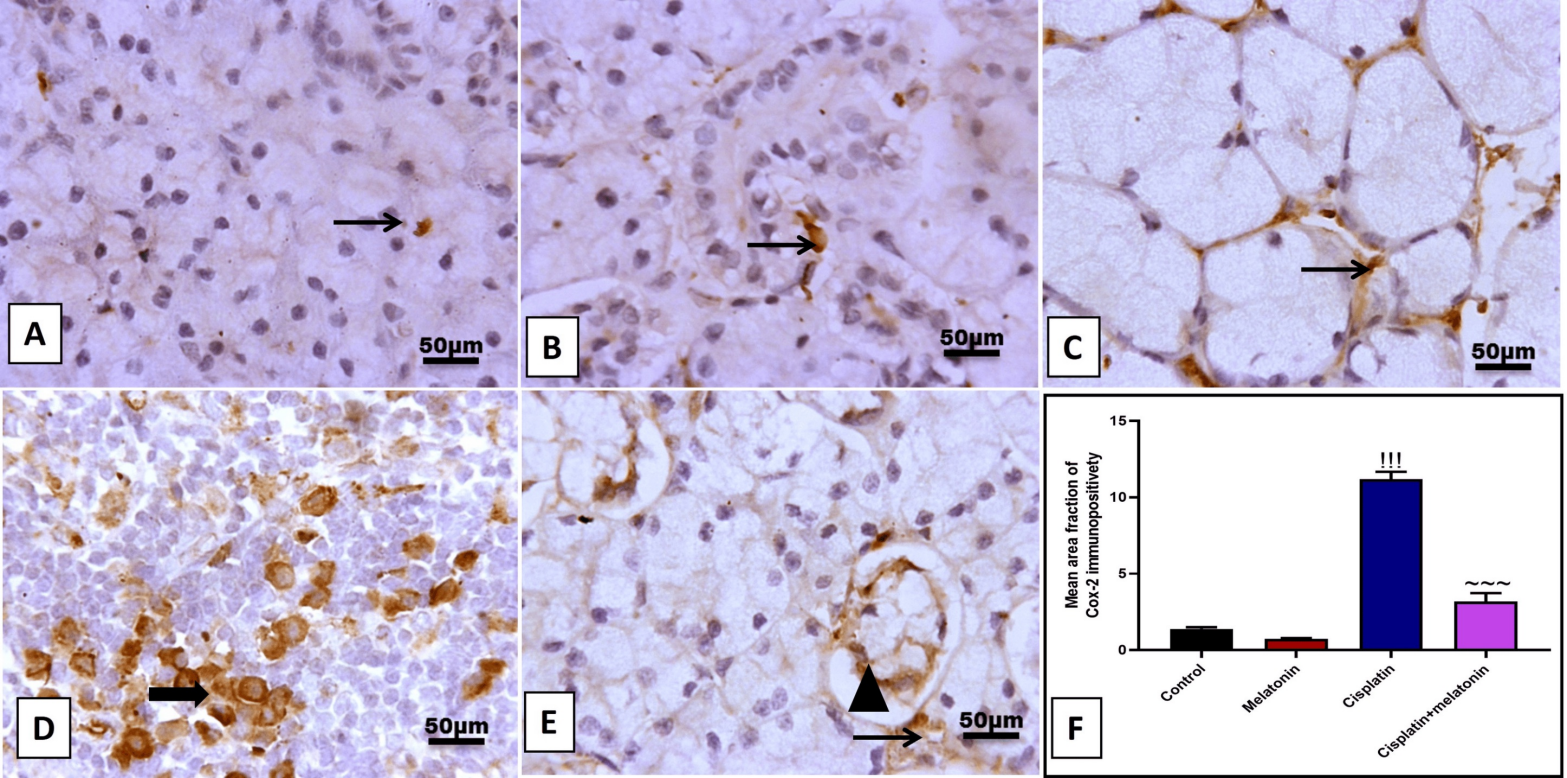
Representative IHC of Cox-2 expression in submandibular gland sections of different treatment groups. (A) Normal group showing few immunoreactivities for Cox-2 in peri-glandular cells. (B) Melatonin group showing few immunoreactivities for Cox-2 in interstitial cells. (C, D) Cisplatin group revealing moderate to high immunoreactivity for Cox in peri-glandular cells with cytoplasmic expression in invading cellular mass. (E) Cisplatin+melatonin showing minimal immunoreactivity for Cox-2 in peri-glandular cells. Image magnification: 400x; arrowhead: positive glandular epithelial cells; thick arrow: positive cellular mass; thin arrow: positive interstitial cells. (F) Quantitative analysis of Cox-2 immunostaining (% area). Data were expressed in mean±SD. !!! indicates p < 0.001 significant versus control rats; ~~~ indicates p < 0.001 significant versus cisplatin group. IHC: immunohistochemistry

**Figure 5:**
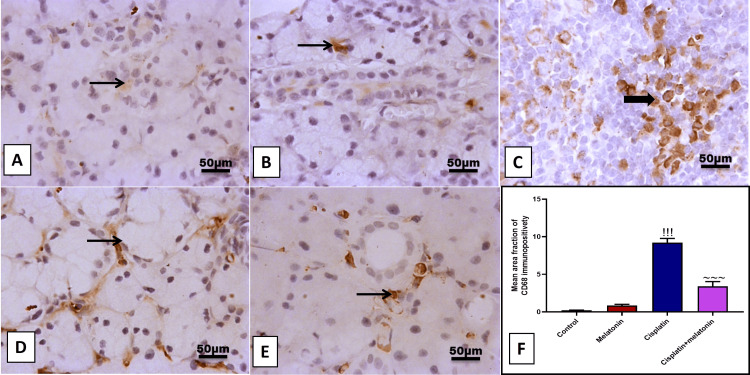
Representative IHC of CD68 expression in submandibular gland sections of different treatment groups. (A, B) Normal and melatonin groups showing few immunoreactivities for CD68 in peri-glandular cells. (C, D) Cisplatin group showing moderate to high immunoreactivity for CD68 in peri-glandular cells and invading cellular mass. (E) Cisplatin+melatonin showing minimal to mild immunoreactivity for CD68 in peri-glandular cells. Image magnification: 400x; thick arrow: positive cellular mass; thin arrow: positive interstitial cells. (F) Quantitative analysis of CD68 immunostaining (% area). Data were expressed in mean±SD. !!! indicates p < 0.001 significant versus control rats; ~~~ indicates p < 0.001 significant versus cisplatin group. IHC: immunohistochemistry

Cisplatin significantly (p < 0.001) increased submandibular mRNA of NF-κB inflammatory transcription factor (Figure 3A), and protein level of proinflammatory TNF-α, IL-6, IL-1β (Figures 3B-3D), with immunoexpression of Cox-2 proinflammatory mediator (Figures 4C, 4D) by 349%, 334.7%, 456%, 502.2%, 810.8% respectively compared to control group (Figures 3A-3D and Figures 4A, 4B). However, melatonin administration significantly (p < 0.001) suppressed the inflammatory markers NF-κB, TNF-α, IL-6, IL-1β, and Cox-2 by 62.1%, 63.6%, 64.4%, 56.9%, 284.1% respectively. Further, examining CD68 which is considered a marker of macrophage infiltration, the cisplatin group showed a significant (p < 0.001) positive brown immunohistochemical staining of CD68 by 418.6% (Figures 5C, 5D, 5F) compared to the control group (Figures 5A, 5B), reflecting the infiltration of the peri-glandular region and cellular mass with macrophages. Nevertheless, macrophages significantly (p < 0.001) reduced by 36.9% in peri-glandular cells of the combined group (Figures 5E, 5F) from the cisplatin group.

Effect of melatonin administration on cisplatin-induced SMG fibrosis

**Figure 6:**
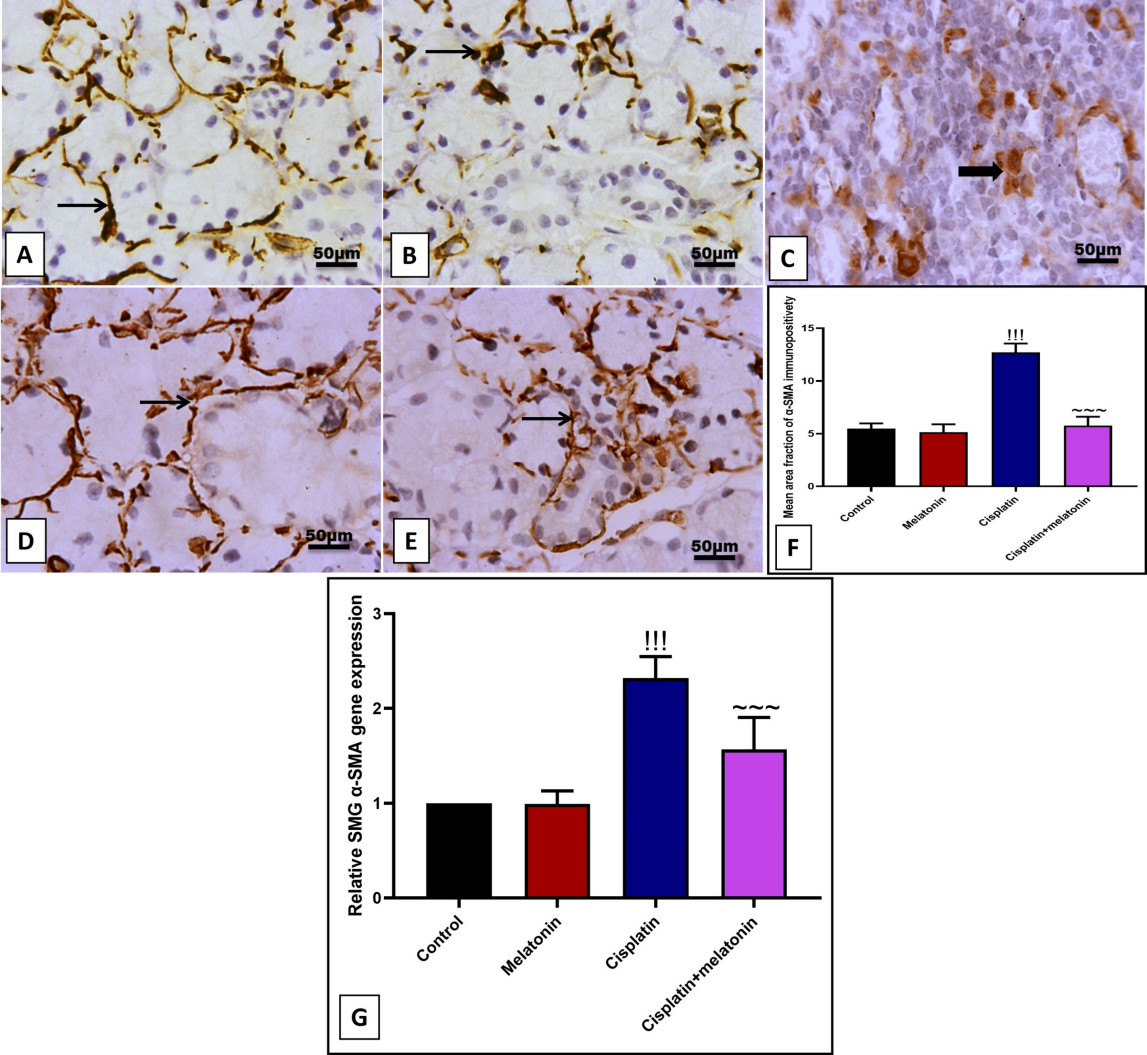
Representative IHC of α-SMA expression in submandibular gland sections of different treatment groups. (A) Normal group showing mild to moderate positive expression of fibril α-SMA around the glandular acini. (B) Melatonin group showing mild to moderate immunostained myocytes. (C, D) Cisplatin group showing high positive nuclear and cytoplasmic staining of invading cells with fibril expression around the dilated gland. (E) Cisplatin+melatonin showing moderate immunostaining in peri-glandular cells. Image magnification: 400x; thin arrow: positive interstitial tissue; thick arrow: positive inflammatory cell. (F) Quantitative analysis of α-SMA immunostaining (% area). (G) Gene expression of α-SMA. Data were expressed in mean±SD. !!! indicates p < 0.001 significant versus control rats; ~~~ indicates p < 0.001 significant versus cisplatin group. IHC: immunohistochemistry; α-SMA: α-smooth muscle actin

**Figure 7:**
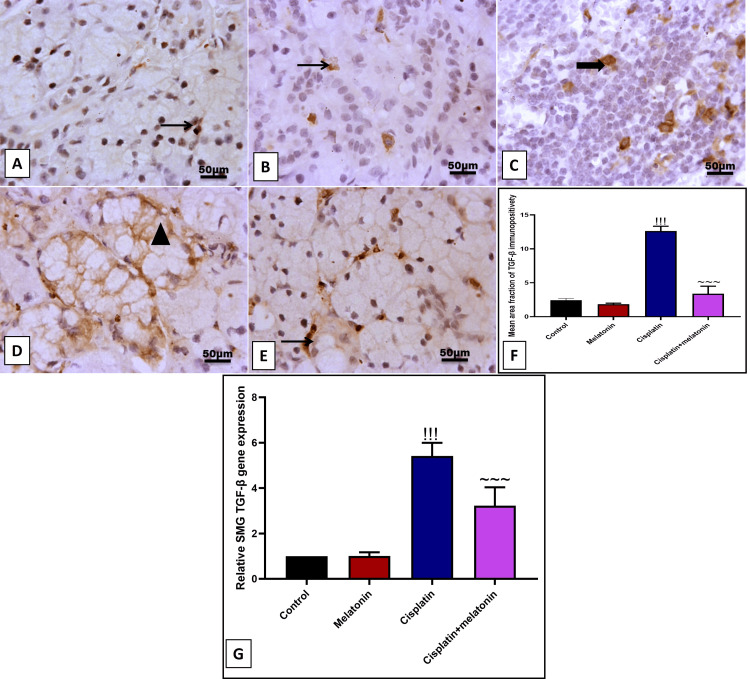
Representative IHC of TGF-β expression in submandibular gland sections of different treatment groups. (A, B) Normal and melatonin groups showing few nuclear immunoreactivities for TGF-β in glandular and peri-glandular cells. (C, D) Cisplatin group showing moderate nuclear with cytoplasmic immunoreactivity for TGF-β in glandular cells and invading cellular mass. (E) Cisplatin+melatonin showing minimal to mild nuclear immunoreactivity for TGF-β in peri-glandular cells. Image magnification: 400x; arrowhead: positive glandular epithelial cells; thick arrow: positive cellular mass; thin arrow: positive interstitial cells. (F) Quantitative analysis of TGF-β immunostaining (%area). (G) Gene expression of α TGF-β. Data were expressed in mean±SD. !!! indicates p < 0.001 significant versus control rats; ~~~ indicates p < 0.001 significant versus cisplatin group. IHC: immunohistochemistry; TGF-β: transforming growth factor beta

Our results revealed a significant (p < 0.001) increase in immunoexpression of brown-stained invading cells and gene expression of α-SMA by 232.5% and 232% (Figures 6C, 6D, 6F, 6G). Likewise, immunoexpression of glandular cells and gene expression of TGF-β by 523.6% and 540% (Figures 7C, 7D, 7F, 7G) compared to control groups (Figures 6A, 6B and Figures 7A, 7B). In contrast, melatonin significantly (p < 0.001) decreased fibrotic markers α-SMA and TGF-β either at the level of the protein by immunoexpression and at the level by mRNA by 45.2%, 67.2%, 26.8%, 59.5% respectively (Figures 6E-6G and Figures 7E-7G) in comparison to cisplatin group.

Protective effect of melatonin against cisplatin-induced SMG apoptosis

**Figure 8:**
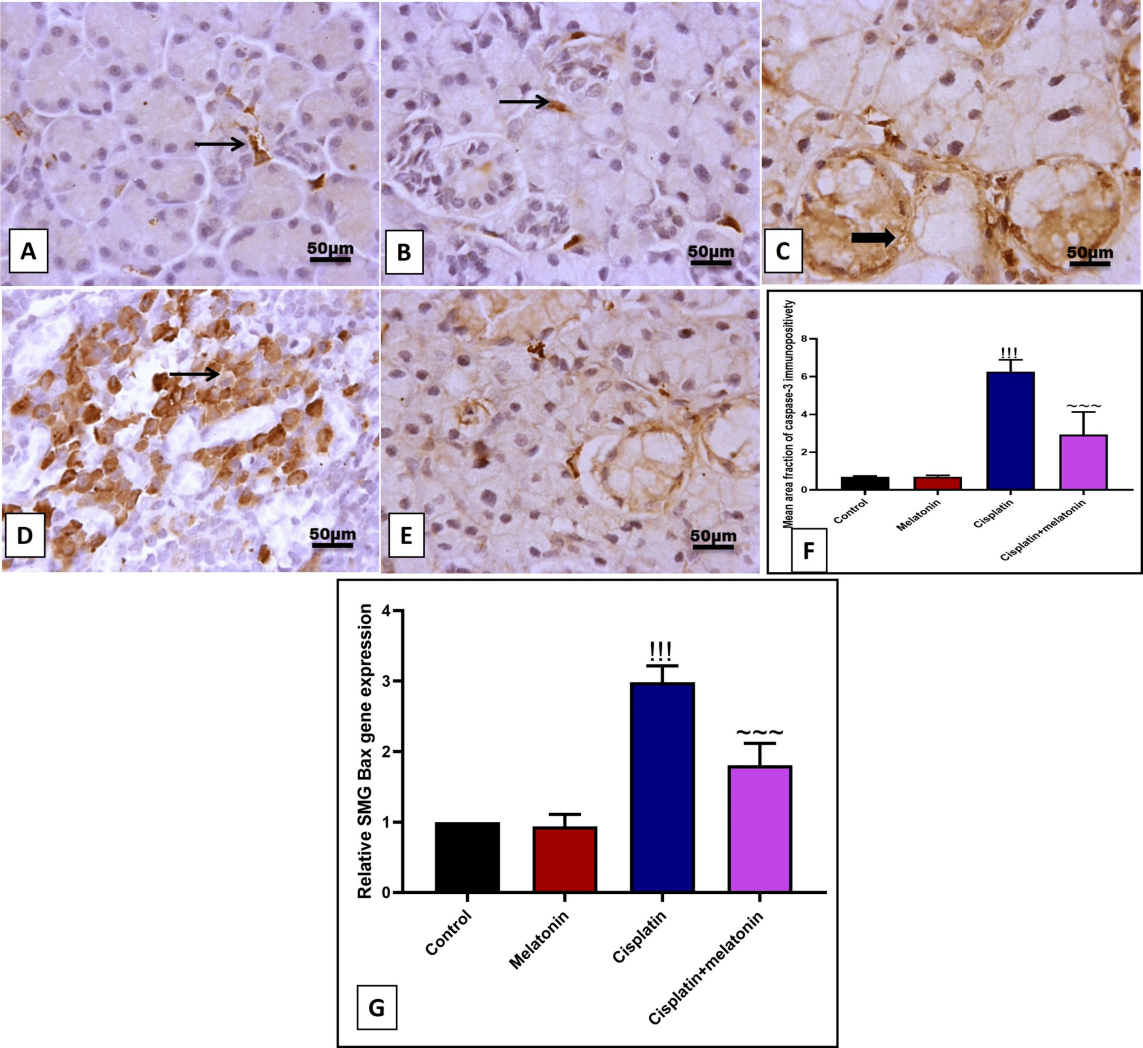
Representative IHC of caspase-3 expression in submandibular gland sections of different treatment groups. (A) Normal group showing few immunoreactivities for caspase-3 in peri-glandular cells. (B) Melatonin group showing few immunoreactivity for caspase-3 in interstitial cells. (C, D) Cisplatin group showing high immunoreactivity for caspase-3 in the cytoplasm of glandular acini with cytoplasmic expression in invading cellular mass. (E) Cisplatin+melatonin showing minimal immunoreactivity for caspase-3 in peri-glandular cells. Image magnification: 400x; thick arrow: positive glandular epithelial cells; thin arrow: positive cellular mass and interstitial cells. (F) Quantitative analysis of caspase-3 immunostaining (% area). (G) Gene expression of α TGF-β. Data were expressed in mean±SD. !!! indicates p < 0.001 significant versus control rats; ~~~ indicates p < 0.001 significant versus cisplatin group. IHC: immunohistochemistry; TGF-β: transforming growth factor beta; SMG: submandibular gland; Bax: Bcl-2-associated X protein

Immunohistochemical examination of the SMG section of the cisplatin group with apoptotic marker caspase-3 revealed a significant (p < 0.001) increase in the brown color in glandular acini, as well as an increase in the mRNA of proapoptotic Bax by 907.2% and 298% (Figures 8C, 8D, 8G) compared to the control group. Likely, melatonin administration significantly (p < 0.001) decreased submandibular cell apoptosis by decreased immunoexpression percent of apoptotic caspase-3 cells and gene expression of Bax by 46.6% and 60.4% (Figures 8E, 8G) in comparison to cisplatin group.

## Discussion

Cancer is the most common cause of death all over the world [16]. Cisplatin is the most potent of the standard chemotherapeutic medications for cancer treatment [17]. The current investigation aims to elucidate the degenerative consequences of chemotherapy on the submandibular salivary gland of rats.

Oxidative stress has the potential to disrupt normal biological processes and initiate cellular death in addition to causing DNA damage [1]. In both in vivo and in vitro studies, it was illustrated that cisplatin induces the excessive overproduction of free radicals [18]. Free radicals, often referred to as ROS, exhibit a high reactivity towards various biological components, leading to the initiation of a series of oxidation and reduction events. Under specific circumstances, the process of oxidative stress might potentially change the structure and function of mitochondria in normal cells, causing organ malfunction [1].

Cisplatin induces degeneration of the submandibular salivary glands, which leads to alternations in SMGs acini and ductal cells. These changes may explain the clinical manifestations of cancer patients. As this gland is responsible for saliva production, cisplatin leads to disturbance in salivary secretions that deteriorate the condition of oral tissues [19]. Both normal and cancer cells are affected by chemotherapy. Salivary glands are easily damaged. It is essential to protect healthy salivary glands against the cytotoxic effects of cisplatin [20]. This study aimed to examine melatonin's ameliorative effect against cisplatin submandibular damage.

In this case, cisplatin is frequently used as an effective chemotherapeutic medication for treating a wide variety of cancers, by attaching to cellular DNA [21] or mitochondrial DNA [22]. Cisplatin can trigger cytotoxic changes that ultimately result in apoptosis or programmed cell death [22,23]. In addition, it promotes the generation of ROS and subsequently leads to an increase in lipid peroxidation and Ca2+ influx, both of which might result in apoptosis [24].

In this study, H&E-stained sections of the SMG revealed marked degeneration in the SMG including dilatation in mucosal acini with replacement of acinar architectures with inflammatory cell infiltration. Similar alterations were detected in the SMGs of cisplatin-treated rats [25]. Its potential is due to induced oxidative stress, followed by accumulation of ROS and lipid peroxidation, which promotes severe cytotoxic effects via the opening of the permeability transition pore complex [26].

Cisplatin causes DNA plastination and cytotoxic alterations that may result in apoptosis [5]. A significant elevation in the area percentage of caspase-3 in the cisplatin group. This observation was made by Elgamily and Denewar [26] in parotid gland cisplatin-treated rats.

As regards the α-SMA immunostaining, this immune reaction showed the degree of myoepithelial cell (MEC) proliferation [27]. MECs, in normal circumstances, line the periphery of the acini and ducts in salivary glands and surround them with their processes [28]. In the current study, there was a notable increase in the area percentage of α-SMA immunostaining in the cisplatin group compared to all other groups. This revealed that cisplatin affected the structural integrity of MECs around both acini and ducts. Such findings support other previous studies as Abdel-Daim et al. [29] and Maghmomeh et al. [30] who noticed increased α-SMA protein levels in the kidneys of cisplatin-treated rats as well as in Nasralla and Mogeda's study, [31] who recorded an increase in α-SMA expression in the myocardium of cisplatin-treated rats. Mousa [32] explained that the change in morphology and proliferation of MECs occur during acinar cell atrophy and regeneration, as in a parenchymal injury, with subsequent increase in their size and number to increase the secretory function of these cells.

Regarding the cisplatin group in this study, there was an increase in apoptosis in comparison with all other groups, which appeared in the form of a marked positive reaction for caspase three antibodies. Mubarak and Ali [33] reported that during the examination of Bax in the parotid salivary glands of the rat, there was intense Bax immunostain in the cisplatin group. Conklin [34] explained increased apoptosis by free radicals produced by cisplatin that destroy the cellular components.

In our study, melatonin administration with cisplatin significantly decreased submandibular lipid peroxidation marker MDA and increased the level of submandibular antioxidant enzyme SOD and GSH. It was reported that melatonin offers a powerful antioxidant function by scavenging free radicals [35]. The antioxidant property of melatonin explains its protective effect on SMG histology.

In the present study, coadministration of melatonin with cisplatin exhibits an anti-inflammatory effect in the form of a marked decrease in the nuclear level of NF-κB with a subsequent decrease in Elisa level of proinflammatory mediators TNF-α, Il-6, IL-1β. This result is supported by the study done by Yapislar et al. [36] who reported that melatonin significantly decreased the level of the inflammatory markers in type 2 diabetic rats. Also, melatonin decreased the immunoexpression of infiltered positive CD68 macrophages, in line with the previous work done by Balsak et al. [37] who reported that melatonin decreased inflammatory marker CD68 in ischemia-reperfusion of ovarian rats. Perhaps melatonin would be anti-inflammatory because it reduces free radical-induced cellular damage. This, in turn, would secondarily reduce the inflammation, which was started by free radicals. Alternatively, it may just be a matter of dose and duration of the treatment.

Respond

The administration of melatonin with cisplatin has been found to have significant implications. It markedly decreases submandibular fibrosis, as evidenced by a decrease in immunostaining and gene expression of TGF-β and α-SMA, a result that is in line with the previous study conducted by Kim et al. [38]. Moreover, melatonin has been found to markedly decrease the number of immunostained caspase-2 positive apoptotic cells, and gene expression of Bax apoptotic marker. This finding aligns with the previous work of Yousif et al. [39], who reported an antiapoptotic effect of melatonin in rats receiving anticancer drugs, as evidenced by a decrease in the serum level of caspase-3. These implications offer hope for the potential use of melatonin in mitigating the side effects of anticancer drugs.

Limitations of the study

The present study, in our mind, is the first one to discuss the protective effect of melatonin against cisplatin-induced submandibular destruction in experimental rats. Although this study focuses on the ameliorative antioxidative power of melatonin with its subsequent inhibition of submandibular inflammation, fibrosis, and apoptosis, further work is needed for more explanation for the definite mechanism of the harmful effect of cisplatin against the SMG, such as autophagy, pyroptosis, and necroptosis.

## Conclusions

The results of our study demonstrated that melatonin administration prevents cisplatin-induced submandibular inflammation, fibrosis, and apoptosis by decreasing the level of NF-κB, IL-6, IL-1β, TNF-α, TGF-β, α-SMA, caspase-3, and Bax respectively. This effect can be attributed to its strong antioxidant character. This finding opens the door for its clinical use against cisplatin-induced submandibular damage.
